# 外泌体在神经退行性疾病中的作用研究进展

**DOI:** 10.3724/SP.J.1123.2024.10035

**Published:** 2025-05-08

**Authors:** Caiting BU, Xuedong ZHU, Qianying ZHANG, Wenya SHAO

**Affiliations:** 1.福建医科大学公共卫生学院预防医学系, 福建 福州 350122; 1. Department of Preventive Medicine, School of Public Health, Fujian Medical University, Fuzhou 350122, China; 2.福建省环境因素与肿瘤重点实验室, 福建 福州 350122; 2. Fujian Provincial Key Laboratory of Environmental Factors and Cancer, Fuzhou 350122, China

**Keywords:** 外泌体, 神经退行性疾病, 生物标志物, 药物载体, exosomes, neurodegenerative diseases, biomarkers, drug microcarrier

## Abstract

外泌体是一种由细胞分泌的纳米级脂质双层囊泡,携带蛋白质、核酸、脂类等重要生物活性分子,广泛存在于血液、脑脊液等体液中。它们通过内吞作用、配体-受体相互作用或直接膜融合等多种机制,将生物活性分子传递给靶细胞,在细胞间通讯中发挥着至关重要的作用。作为天然的药物传递系统,外泌体具有生物相容性、高透过性、靶向性和含天然治疗分子等优势,能够提高药物递送的精确性和疗效,从而成为向中枢神经系统递送药物的理想载体。因此,外泌体在中枢神经系统疾病的诊断与治疗中显示出巨大的潜力。本文系统综述了近年来外泌体在神经退行性疾病中的研究进展,重点探讨了其在疾病发病机制、病程进展、诊断和治疗中的作用,旨在为神经退行性疾病的早期诊断与治疗提供理论依据与研究参考。

## Abstract (en)

Exosomes are nano-sized, lipid bilayer vesicles secreted by cells. They carry essential bioactive molecules, such as proteins, nucleic acids, and lipids, and are widely present in bodily fluids including blood and cerebrospinal fluid. Exosomes transfer bioactive molecules to target cells through various mechanisms, including endocytosis, ligand-receptor interactions, or direct membrane fusion, and play crucial roles in intercellular communication, including facilitating intercellular information exchange, maintaining nerve-cell function, participating in immune responses, and providing nutritional support. Exosomes significantly promote signal transmission and intercellular communication in the central nervous system and are involved in the pathogenesis and development of diseases by participating in the spread of pathological proteins, regulating neuroinflammation, and the deposition of pathological proteins. Therefore, exosomes play key roles in the occurrence and development of neurodegenerative diseases, and their contents, especially proteins and miRNAs, are specific for given pathological and physiological states and are relatively stable during extraction and analysis. Hence, exosomes are ideal tools for diagnosing diseases, staging their courses, and assisting prognosis. This article further explores exosomes derived from blood, saliva, urine, and cerebrospinal fluid as potential diagnostic biomarkers for neurodegenerative diseases. As natural drug-delivery systems, exosomes have the advantages of biocompatibility, ability to cross biological barriers, target specificity, stability, and containing natural therapeutic molecules, which can effectively improve the precision and efficacy of drug delivery and reduce side effects, making them an ideal carrier for delivering drugs to the central nervous system. Therefore, exosomes hold great potential in the diagnosis and treatment of central nervous system diseases. This article systematically reviews the latest advances in exosome research directed toward specific neurodegenerative diseases, focusing on their roles played in disease pathogenesis, progression, diagnosis, and treatment, with the aim of providing theoretical support and a reference for the early diagnosis and treatment of these diseases.

外泌体(exosomes)是一种直径约为30~200 nm的双层磷脂膜囊泡,能通过细胞间隙或体液传递信息,调控细胞功能,并参与疾病进程^[1]^,是疾病早期诊断、鉴定及预后的生物标志物有效来源^[2]^,且可透过血脑屏障,有望成为神经退行性疾病预防和诊疗的新突破口。因此,近年来外泌体在神经退行性疾病中的作用引起了广泛关注。

随着人口老龄化的加剧,神经退行性疾病已经成为一个日益严重的全球公共卫生问题。这类疾病涵盖了阿尔茨海默病(Alzheimer’s disease, AD)、帕金森病(Parkinson’s disease, PD)、亨廷顿病(Huntington’s disease, HD)、肌萎缩侧索硬化症(amyotrophic lateral sclerosis, ALS)以及多发性硬化症(multiple sclerosis, MS)等。这类疾病通常伴随蛋白质异常聚集的病理机制^[3,4]^,但早期症状通常不具特异性,易与其他病症混淆,加之缺乏有效的生物学标志物,早期诊断极具挑战性。同时,中枢神经系统的血脑屏障(blood-brain barrier, BBB)具有高度选择性,限制了药物的治疗效果^[5]^。由于外泌体携带多种生物标志物,在神经退行性疾病的诊断方面显示出巨大潜力。另外,作为细胞外纳米级囊泡,外泌体能穿过生物屏障并直接靶向特定细胞,递送治疗分子,为神经退行性疾病的治疗提供了新策略^[6]^。本文对近年来外泌体在神经退行性疾病中的作用进行了总结和展望。

## 1 外泌体的生物学特性

外泌体是一类由细胞分泌至细胞外空间的膜性囊泡,通过内吞作用、配体-受体相互作用或直接膜融合的方式,将生物活性分子传递给靶细胞,从而在细胞间通讯中扮演重要角色^[7]^。

外泌体的内容物丰富多样,包括来源细胞特异性的mRNA和非编码RNA(microRNA、long non-coding RNA等),这些遗传物质能够影响受体细胞的转录组和蛋白质组状态,参与基因表达的调控。例如,miR-132和miR-21能够调节血管完整性^[8]^、神经元与巨噬细胞间的通讯和炎症反应^[9]^。外泌体还包含DNA片段,这些片段在细胞内稳态、肿瘤发生、治疗和免疫记忆中发挥作用。外泌体蛋白质种类繁多,包括膜蛋白、胞质蛋白、内质网和高尔基体相关蛋白等。外泌体膜蛋白主要分为两大类:一类是普遍存在的标记蛋白,如四次跨膜蛋白(如CD9、CD63、CD81和CD82)、Tsg101、Alix和flotillin-1^[10]^。另一类是特定细胞来源的外泌体特有膜蛋白,例如抗原提呈细胞外泌体中的MHC-Ⅱ和CD86。此外,外泌体还可能含有毒性蛋白聚集物,如α-突触核蛋白(α-synuclein, α-syn)、Tau蛋白、β-淀粉样蛋白(amyloid β-protein, Aβ)和朊病毒致病蛋白^[11]^,这些成分在神经退行性疾病中具有病理意义。

外泌体的形成过程涉及细胞质膜的两次内陷及多泡小体(multivesicular bodies, MVBs)的形成。细胞质膜的第一次内陷形成早期内体(early-sorting endosome, ESE)。早期内体在质子泵介导的酸化作用下成熟,转变为晚期内体(late-sorting endosome, LSE)^[12]^,并进一步转化为MVBs。MVBs一部分与自噬体或溶酶体融合而降解,另一部分在鸟苷三磷酸酶家族中Rab酶的调控下与细胞膜融合,并通过胞吐作用释放外泌体^[13]^。外泌体在释放到细胞外环境后,能够通过多种途径与靶细胞相互作用,包括直接与靶细胞膜融合、受体介导的内吞作用(包括吞噬作用和胞饮作用),以及通过外泌体表面配体与靶细胞受体的相互作用(图1),使得外泌体在细胞间通讯、信号传递和多种生物学过程中发挥着关键作用。

**图1:**
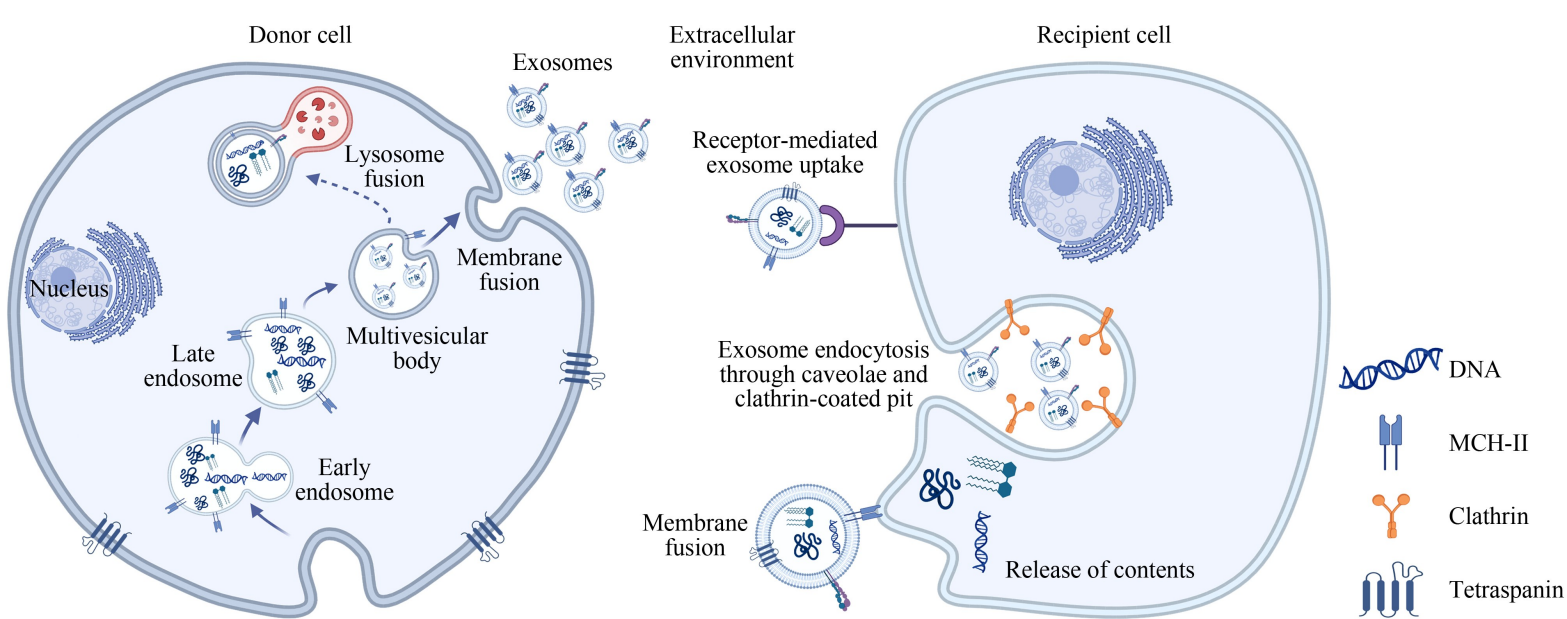
外泌体的产生与靶细胞摄取外泌体的过程

外泌体的分泌过程受到精密调控,主要涉及两种途径:转运必需内体分选复合物(endosomal sorting complexes required for transport, ESCRT)依赖途径和非ESCRT依赖途径,确保了外泌体内容物的特定分选^[14]^。研究发现,Rab蛋白亚家族成员,如Rab11a、Rab27A/B、Rab37、Rab39等小分子GTP结合蛋白^[15,16]^以及介导囊泡与靶膜融合的可溶性NSF附着蛋白受体(soluble NSF attachment protein receptor, SNARE)复合物^[17]^在调控外泌体的分泌时间和分泌量方面发挥着至关重要的作用。

## 2 不同细胞来源的外泌体在中枢神经系统中的功能

在中枢神经系统中,外泌体能通过内吞、受体-配体结合、附着等方式与靶细胞相互作用,发挥多种生物学功能,包括细胞间通讯、维持髓鞘化、抗原提呈和提供神经元营养支持等。不同细胞来源和微环境的外泌体成分和功能存在差异^[18]^。

小胶质细胞来源的外泌体在中枢神经系统(central nervous system, CNS)中起到多种生物学调控作用,能够影响神经炎症、突触功能、神经保护以及神经元凋亡。(1)神经炎症调控:小胶质细胞是CNS的免疫监测细胞,M1型小胶质细胞外泌体富含炎症因子(如TNF-α、IL-6、IL-1β),可激活周围细胞并加剧炎症,导致神经元受损。相对地,M2型小胶质细胞外泌体携带抗炎因子(如IL-10),在神经系统中有助于炎症的抑制,促进受损组织修复。这种双向调控使小胶质细胞在神经炎症中的作用变得更加复杂。(2)突触修剪:外泌体在突触功能调控和病理性修剪中同样重要。研究显示^[19]^,小胶质细胞外泌体含有补体成分,可能通过标记神经元突触,促进病理条件下的突触剥离,这种突触修剪功能可能与神经退行性疾病中的突触损失和功能异常有关。(3)神经保护与细胞通讯:Liu等^[20]^发现活化的小胶质细胞可通过外泌体向多巴胺能神经元传递环状RNA ZNRF,改善百草枯介导的神经元凋亡,延缓PD进程。此外,外泌体的转运能力使得小胶质细胞能够将信号传递至大脑的远端区域,维持CNS的细胞间通信。(4)病理蛋白的传递和神经元凋亡:小胶质细胞外泌体能携带特定的病理性蛋白质,如α-syn,传递给邻近的神经元,进而在神经元之间引发病理性聚集和传播。这一特性在PD、AD等神经退行性疾病中尤为明显。

近年来,干细胞来源的外泌体同样引起了广泛关注,展现出在神经系统疾病治疗中的重要应用价值,包括抑制氧化应激、减轻炎症反应、调节小胶质细胞极化、促进血管生成和神经发生等。间充质干细胞来源的外泌体(MSC-Exos)能够促进大脑血管生成和神经发生,有利于大脑组织重塑,并通过激活单磷酸腺苷(adenosine monophosphate, AMP)依赖的蛋白激酶磷酸化和下调JAK2/STAT3/NF-κB信号通路来改善神经元凋亡^[21]^。牙髓干细胞来源的外泌体能通过抑制HMGB1/TLR4/MyD88/NF-κB信号通路减轻神经炎症和脑损伤^[22]^。此外,MSC-Exos能减少星形胶质细胞的活化,抑制炎症因子表达,减轻AD模型的神经炎症,并通过上调miR-21的水平恢复突触功能,改善AD小鼠的学习和记忆能力。Chen等^[23]^的研究发现,MSC-Exos能够穿过血脑屏障到达黑质,抑制多巴胺能神经元凋亡,并上调纹状体中的多巴胺水平,展现出治疗PD的潜力。

### 2.1 外泌体与阿尔茨海默病的发生发展

AD的主要病理特征是Aβ和磷酸化Tau蛋白的异常聚集。外泌体在Aβ的生成、分泌、聚集和传播过程中扮演着关键角色。它们不仅通过提供酸性环境促进淀粉样前体蛋白(APP)剪切形成Aβ,而且富含糖脂和脂筏的外泌体还为Aβ纤维的形成提供了有利环境,并促进了Aβ在细胞间的传播。此外,外泌体能够通过硫酸肝素蛋白聚糖、凝集素、免疫球蛋白和四次跨膜蛋白等多种途径被受体细胞摄取,这会引发受体细胞内体-溶酶体系统及自噬功能的失调,增加细胞毒性寡聚物的生成和淀粉样蛋白的释放,最终促进斑块的形成^[24]^,如在AD患者脑组织的淀粉样斑块中已发现外泌体标记蛋白^[25]^。

星形胶质细胞来源的外泌体在正常情况下有助于维持神经元存活和抑制神经炎症。然而,随着AD的进展,Aβ对星形胶质细胞的激活可能导致这些细胞分泌具有促凋亡特性的外泌体^[26]^,加剧神经元的损伤。此外,在AD早期,小胶质细胞通过释放外泌体吞噬Aβ,减少Aβ沉积;而在AD后期,小胶质细胞外泌体会释放pro-IL-1β、caspase-1和毒性Aβ,进一步促进神经炎症和神经损伤^[27]^。

外周巨噬细胞源的外泌体也参与AD的炎症过程。研究发现,这些外泌体能够影响小胶质细胞的极化状态,使其偏向M1促炎表型,增加AD的炎症负担,进而增加AD的发病风险^[28]^。上述发现表明,外泌体在AD病程中不仅作为蛋白的载体影响病理扩散,还在不同细胞之间传递信号,调控神经炎症,从而为AD的发生与发展提供了新的理解和研究方向。

### 2.2 外泌体与帕金森病的发生发展

PD是一种以黑质纹状体多巴胺能神经元选择性丢失为特征的进行性神经退行性疾病。α-syn的错误折叠和原纤维聚集是PD病理进程的关键事件,能通过多种途径如神经炎症、氧化应激和细胞自噬^[29,30]^促进疾病发展。Stuendl等^[31]^的研究表明,PD患者的脑脊液外泌体中存在错误折叠的α-syn寡聚体,这些寡聚体可作为“种子”诱导健康神经元中α-syn的聚集,这种聚集过程可能沿着神经元纤维传播,导致神经元损伤和死亡,从而加速PD的进展。因此,外泌体中的α-syn寡聚物比游离形式更具神经毒性^[32]^,且更容易被细胞摄取。正常生理状态下,小胶质细胞能够吞噬并清除错误折叠的α-syn,但PD患者血浆来源的外泌体会降低小胶质细胞内负责细胞内环境酸化的V1G1蛋白表达,损害溶酶体的酸化功能,使得溶酶体酶活性下降,进而影响溶酶体蛋白的降解功能,导致小胶质细胞中α-syn进一步聚集和炎症激活^[33]^。

自噬过程在清除错误折叠的α-syn中至关重要,但当溶酶体功能受损时,外泌体介导的α-syn释放和传播进一步增加。例如,抑制自噬-溶酶体通路的药物Bafilomycin会增加α-syn外泌体的释放^[34]^,而通路激动剂雷帕霉素则减少α-syn外泌体的释放,对神经元起到保护作用^[35]^。另外,NOD样受体热蛋白结构域相关蛋白3(NOD-like receptor family, pyrin domain containing 3,NLRP3)炎症小体能够促进外泌体的释放,并通过激活TLR4/MyD88信号通路,加速α-syn的扩散与沉积^[36]^。

此外,外泌体中的miRNA也在PD病程中扮演重要角色。miR-7和miR-153的缺失导致无法抑制*SNCA*基因mRNA的转录^[37]^以及α-syn的上调和聚集,加剧多巴胺能神经元损伤。抗氧化蛋白DJ-1的减少增加了α-syn毒性,而外泌体携带的miR-4639-5p负调节DJ-1^[38]^,加剧氧化应激和神经元死亡。外泌体富亮氨酸重复激酶基因(leucine-rich repeat kinase 2, *LRRK2*)突变是PD发病机制中常见的遗传因素,其突变增加了MVBs内磷酸化α-syn的含量^[39]^,从而改变了外泌体内蛋白质的组成,导致细胞通讯和信息交流的病理性失调。Chang等^[40]^的研究指出,外泌体能够通过肿瘤坏死因子依赖方式诱导神经元凋亡。这些发现进一步证实了外泌体在PD病理发生发展中的重要作用,并提示外泌体可能成为PD靶向治疗的潜在目标。

### 2.3 外泌体与亨廷顿病的发生发展

HD是一种常染色体显性遗传病,其病理基础在于Huntingtin蛋白(HTT)基因中的CAG重复序列扩增,当重复次数超过36次时,会导致含有多聚谷氨酰胺(poly Q)的HTT蛋白形成包涵体,损害包括神经元、星形胶质细胞和小胶质细胞在内的各种脑细胞。研究显示,外泌体能够运输异常的HTT蛋白及RNA。此外,Zhang等^[41]^使用人胚胎肾293T细胞作为实验中外泌体的供体细胞,发现释放的外泌体中包含了poly Q和CAG重复RNA,表明外泌体可能具有促进HD病理扩散的潜力。Wang等^[42]^通过对HD模型小鼠的纹状体和HD患者的大脑额叶皮质及尾状核进行研究,发现50%~60%的差异表达miRNA存在于外泌体中,可能在HD的发病机制中发挥重要作用。

### 2.4 外泌体与肌萎缩侧索硬化症的发生发展

ALS的病理机制与多种蛋白的异常折叠和聚集密切相关,包括朊病毒样蛋白、铜-锌超氧化物歧化酶1(superoxide dismutase 1, SOD 1)和TAR DNA结合蛋白43(TDP-43)。Silverman等^[43]^从ALS小鼠脑组织中分离出CNS衍生的外泌体,发现这些外泌体含有ALS致病性蛋白,证实了错误折叠的SOD 1蛋白可以通过外泌体依赖和独立的途径在细胞间转移。Nonaka等^[44]^用携带难溶性TDP-43蛋白聚集物的外泌体处理表达正常可溶性TDP-43蛋白的细胞,观察到难溶性TDP-43聚合物朊病毒样传播,引起细胞内TDP-43蛋白的异常积聚。且相较于通过非外泌体途径分泌的TDP-43蛋白,经外泌体分泌的TDP-43蛋白更易被邻近细胞摄取,并展现出更强的细胞毒性。

在遗传因素方面,约4%~5%的家族性ALS和部分散发性ALS患者是由*FUS*基因突变所致。Kamelgarn等^[45]^的研究揭示,FUS蛋白能够通过外泌体途径被分泌,这表明外泌体可能参与了突变FUS蛋白在细胞间的传播,从而促进了病情进展。*C9orf72*基因内含子区六核苷酸(GGGGCC)重复片段拷贝数的异常扩增(> 30次)是已知的ALS致病基因中最常见的突变,可编码5种二肽重复蛋白(dipeptide repeat proteins, DPRs)。*C9orf72*基因阳性的ALS患者的CNS中有DPR包涵体形成,产生细胞毒性,影响细胞功能。Westergard等^[46]^发现部分DPRs可通过外泌体进入周围细胞,导致病理改变的扩散。

## 3 外泌体在神经退行性疾病诊断中的研究进展

外泌体参与神经细胞间的通讯,在PD的发生和发展中发挥重要功能。由于其纳米级的尺寸特性,外泌体能够穿透血脑屏障,进入外周循环,从外周血中即可获得。外泌体的内容物(特别是蛋白质和miRNA)在不同病理生理状态下具有特异性,在提取过程中不易受影响,这使得外泌体成为疾病诊断、病程分期和预后评估的理想工具。

### 3.1 外泌体与阿尔茨海默病的诊断

AD的临床诊断目前依赖于症状观察、神经心理测试、腰椎穿刺和神经影像学技术,但尚无公认的金标准。尽管腰椎穿刺有助于AD诊断,其侵入性特点使部分患者望而却步。相比之下,血液中提取的外泌体具有无创性、高灵敏度和高特异性优势。研究表明^[47]^, AD患者外周血神经源性外泌体中的Aβ42、T-tau和P-T181-tau水平显著高于轻度认知障碍(MCI)患者和健康对照组,且这些标志物的水平与脑脊液中对应标志物的水平高度相关,提示血液外泌体携带的生物标志物在AD和MCI的诊断中具有与脑脊液相当的诊断效果。

此外,脑脊液中的外泌体miR-328-3p水平可以区分AD与额颞叶痴呆^[48]^。脑脊液Aβ42、T-tau和P-tau可用于早期诊断,以区分AD轻度认知障碍患者与稳定轻度认知障碍患者^[49]^。神经元源性外泌体中携带的与AD发病相关的标志物,如磷酸化胰岛素受体底物1(phosphorylated insulin receptor substrate 1, P-IRS1)、胱天蛋白酶D(Cathepsin D)和突触蛋白(Synapsin),有望成为AD诊断的生物标志物^[50]^。使用超灵敏免疫检测分析早发性和晚发性AD患者以及健康老年人血浆外泌体^[51]^发现,神经营养因子(BDNF、VEGF、TGF-β)在早发性和晚发性AD患者中均升高,与认知功能和灰质体积相关;而退行性标志物(GFAP、NfL)在早发性AD患者中显著升高,与病情严重程度相关,为AD诊断和分期提供了潜在的血液标志物。

### 3.2 外泌体与帕金森病的诊断

目前,PD的诊断主要依赖于神经系统症状和大脑成像技术,但这些方法往往不够精确且诊断过程漫长,从症状出现到确诊大约有12个月的延迟,多数患者在运动症状显著时才被诊断,此时已丢失约70%的多巴胺能神经元^[52]^。因此,为实现早期诊断和疾病进展监测,寻找具有高灵敏度和高可靠性的生物标志物至关重要。

Rani等^[53]^的研究显示,PD患者唾液细胞外泌体中的α-syn寡聚物水平及α-syn寡聚物/总α-syn比值显著升高,其敏感性和特异性优于脑脊液和血液,唾液样本更易获取且避免了溶血污染。非震颤PD患者的血浆神经源性外泌体中α-syn含量高于震颤患者^[54]^,显示其对PD运动与非运动障碍的区分价值。另一项研究显示,血清神经元源性外泌体中的α-syn与聚集蛋白相结合可预测和区分PD与非典型帕金森综合征^[55]^。

Jiang等^[56]^通过无标记定量蛋白质组学分析了PD患者和健康对照组血清中分离的外泌体,发现PD患者的色素上皮因子(pigment epithelium-derived factor, PEDF)、载脂蛋白D(apolipoprotein D, ApoD)等7种蛋白表达水平显著升高,而补体C1q和免疫球蛋白IGLV1-33等蛋白表达水平下降,这些差异表达的蛋白质被证实可作为PD早期诊断的潜在生物标志物。DJ-1蛋白的突变型与早发型PD相关,PD患者血浆中的中枢神经源性细胞外泌体DJ-1蛋白水平以及其与血浆中枢神经源性总DJ-1的比值均显著高于对照组^[57]^。在特发性PD患者中,尿液外泌体中LRRK2蛋白的Ser-1292位点磷酸化水平显著增加,这种现象在男性患者中尤为显著,且其水平与认知功能障碍程度以及日常生活能力的降低程度呈正相关^[58]^。Hadisurya等^[59]^利用尿液外泌体蛋白质组学和磷酸化蛋白质组学分析,成功鉴定和定量了数千种蛋白质和磷酸化蛋白质,并通过机器学习和生物信息学分析,建立了一个包含蛋白PCSK1N、HNRNPA1、pPLA2G4A、pLTB4R、pPRR15等多种生物标志物的诊断面板,这些生物标志物有望成为PD早期诊断的有力工具。此外,1-甲基-4-苯基-1,2,3,6-四氢吡啶(1-methyl-4-phenyl-1,2,3,6-tetrahydropyridine, MPTP)诱导的PD模型小鼠中血清及脑组织外泌体的代谢物分析揭示了与氧化脂质和胆固醇代谢相关的特征性变化,显示其作为临床前生物标志物的潜力^[60]^。

小胶质细胞外泌体的miR-92a-3p和miR-24-3p水平在PD患者中显著升高,可能作为早期诊断的潜在标志物^[61]^。另外,Manna等^[62]^通过TaqMan技术测定了健康对照者、PD患者和进行性核上性麻痹患者的血清miRNA水平,发现miR-21-3p、miR-22-3p等外泌体miRNA可作为区分PD与进行性核上性麻痹的生物标志物。通过纯化PD患者脑脊液中小胶质细胞/巨噬细胞来源的外泌体,研究发现PD患者脑脊液中的外泌体α-syn含量高于健康对照组^[63]^。与健康对照组相比,PD患者脑脊液外泌体中存在差异表达的miRNA, miR-1和miR-19b-3p的表达水平显著降低,而miR-153、miR-409-3p、miR-10a-5p和let-7g-3p的表达水平显著升高^[64]^,揭示了脑脊液外泌体在PD诊断中的潜在应用价值。

综上所述,外泌体中携带的生物标志物在PD的早期诊断和监测中展现出广阔前景,未来仍需通过临床研究验证其诊断效力,为PD的精准医学提供新靶点。

### 3.3 外泌体与亨廷顿病、肌萎缩侧索硬化症的诊断

Reed等^[65]^发现,在HD患者出现症状前,可以检测到外泌体中miR-135b-3p、miR-140-5p等miRNA水平显著升高。CAG重复RNA和polyQ蛋白片段可以进入外泌体,并且在分化的纹状体细胞系中,含有扩增CAG重复的mRNA比含有正常CAG重复的mRNA更倾向于被纳入外泌体。这一发现表明外泌体中的CAG重复RNA和polyQ蛋白水平可能作为HD进展的生物标志物^[66]^。

Xu等^[67]^在研究ALS患者血清外泌体时发现,miR-27a-3p表达显著下调,显示出作为ALS诊断标志物的潜力。同时,Otake等^[68]^利用高通量测序技术分析了ALS患者和健康人群脑脊液中的外泌体mRNA,识别出543个表达显著差异的基因(如*CUEDC2*),这些基因涉及蛋白质降解、氧化应激和错误折叠等ALS的病理过程。该研究表明,脑脊液中的外泌体mRNA可为ALS的早期诊断提供潜在的生物标志物,同时为理解ALS的病理机制提供了新的视角。

综上,已经报道的外泌体中有望用于神经退行性疾病诊断的潜在生物标志物如表1所示(重点介绍来源于血液、唾液和尿液、脑脊液的外泌体)。

**表1:** 外泌体中有望用于神经退行性疾病诊断的潜在生物标志物

Disease	Body fluid	Potential biomarkers	Sensitivity	Specificity	AUC	Ref.
AD	blood	Aβ42	-	-	0.930	[47]
		T-tau	-	-	0.890	[47]
		P-T181-tau	-	-	0.890	[47]
		P-IRS1, Caspase D, Syn	-	-	-	[50]
		BDNF, VEGF, TGF-β, GFAP, NfL	-	-	-	[51]
	CSF	miR-328-3p	-	-	0.702	[48]
		Aβ42, T-tau, P-tau	-	-	-	[49]
PD	saliva	α-syn oligomer	-	-	-	[53]
	blood	α-syn	0.67	0.71	0.675	[54]
		α-syn interacts with aggregated proteins	0.94	0.96	0.980	[55]
		PEDF, ApoD, C1q, IGLV1-33	-	-	-	[56]
		DJ-1	0.80	0.58	0.703	[57]
		(*S*)-(-)-2-hydroxyisocaproic acid	-	-	1	[60]
		miR-92a-3p, miR-24-3p	-	-	-	[61]
		miR-21-3p, miR-199a-5p, miR-425-5p, miR-483-5p,	0.89	0.90	0.910	[62]
		miR-22-3p, miR-29a-3p				
	CSF	α-syn	-	-	-	[63]
		miR-409-3p	-	-	0.970	[64]
		miR-19b-3p	-	-	0.705	[64]
		miR-10a-5p	-	-	0.900	[64]
	urine	Ser(P)-1292 LRRK2	0.60	0.90	0.788	[58]
		PCSK1N, HNRNPA1, pPLA2G4A, pLTB4R, pPRR15	1	0.69	0.943	[59]
HD	CSF	miR-135b-3p, miR-140-5p	-	-	-	[65]
		CAG Repeat RNA, polyQ protein	-	-	-	[66]
ALS	blood	miR-27a-3p	-	-	-	[67]
	CSF	*CUEDC2*	-	-	-	[68]

AD: Alzheimer’s disease; PD: Parkinson’s disease; HD: Huntington’s disease; ALS: amyotrophic lateral sclerosis; CSF: cerebrospinal fluid; P-IRS1: phosphorylated insulin receptor substrate 1; BDNF: brain-derived neurotrophic factor; VEGF: vascular endothelial growth factor; TGF-β: transforming growth factor-β; GFAP: glial fibrillary acidic protein; NfL: neurofilament light chain; PEDF: pigment epithelium-derived factor; PCSK1N: proprotein convertase subtilisin/kexin type 1 inhibitor; HNRNPA1: heterogeneous nuclear ribonucleoprotein A1; pPLA2G4A: phosphorylated phospholipase A2 group IVA; pLTB4R: phosphorylated leukotriene B4 receptor; pPRR15: phosphorylated proline-rich receptor 15; AUC: area under the curve, refers to the area beneath the receiver operating characteristic curve.

## 4 外泌体与神经退行性疾病的治疗

采用外泌体作为药物传递载体,不仅能实现更加精准的靶向治疗,还能提高局部浓度,进而减少副作用并增强疗效。与传统的药物传递方式相比,外泌体具有显著优点。(1)天然存在:外泌体是细胞自然分泌的产物,因此在体内展现出良好的生物相容性及低免疫原性,不易被巨噬细胞清除;(2)高透过性:外泌体由于其纳米级大小能够通过血脑屏障,将蛋白质、代谢分子和核酸等输送至靶细胞,从而有效调控细胞功能和代谢;(3)靶向性:外泌体表面的特定分子,如蛋白质、脂质和糖类,能识别并结合特定细胞受体,实现精准给药,减少对非靶组织的副作用;(4)稳定性:外泌体的膜结构为其内部分子提供了保护,防止体内酶解和降解;(5)含有天然治疗分子:外泌体不仅作为药物载体,其本身也包含治疗成分。来自间充质干细胞、神经干细胞等细胞的外泌体携带的miRNA和蛋白质具有神经保护作用,能促进神经发生,改善处于疾病状态下的神经功能。

### 4.1 外泌体载药系统的构建与优化

作为一种具有治疗潜力的药物递送载体,外泌体能够通过内源装载和外源装载两种方法有效地将药物成分输送至特定的靶器官、组织和细胞。内源装载通过改变亲本细胞的培养条件实现药物的预装载,而外源装载则涉及对纯化后的外泌体进行物理或化学处理以实现药物的后期装载,包括孵育法、电穿孔法、超声法、挤压法、冻融法等^[69]^。

外泌体的双层膜结构主要由亲脂性脂质构成,这使得脂溶性药物更容易进入并嵌入膜中,实现稳定的封装。适合利用外泌体装载的脂溶性药物包括姜黄素^[70]^、紫杉醇^[71]^、奥拉帕尼^[72]^、云杉醇^[73]^等。水溶性药物由于其亲水性质,通常难以有效地装载到疏水性的外泌体膜中。为了提高水溶性药物如多柔比星的装载效率,研究者们采用了电穿孔和超声等方法来增加外泌体膜的通透性,使药物能够进入外泌体^[71]^。因此,在选择装载方法时,需要权衡药物的性质和装载效率等因素。

与游离药物相比,载药外泌体展现出更好的疗效和更高的生物利用度,这得益于外泌体的工程化表面修饰,包括基因工程、共价修饰和非共价修饰等,这些修饰增强了外泌体的靶向性和生物学功能,使其成为一种精准的药物递送载体。

### 4.2 外泌体与阿尔茨海默病的治疗

目前,市场上针对AD的药物主要在缓解症状、改善认知和提高生活质量方面有效,无法阻止疾病的进展。研究表明,外泌体可能在AD的治疗中发挥独特作用。Wei等^[74]^发现,在AD细胞模型中,miR-223表达下调与细胞凋亡增加和迁移受损相关,而通过与MSC-Exos共培养,可以上调miR-223并通过PTEN-PI3K/Akt途径减少神经细胞凋亡。此外,Ding等^[75]^通过注射含有脑啡肽酶(NEP)和胰岛素降解酶(IDE)活性的MSC-Exos,有效降低了AD模型小鼠大脑中的Aβ斑块沉积,抑制了炎症因子活性并提高了抗炎因子水平,减轻了神经炎症。Chen等^[76]^的研究显示,将MSC-Exos移植到AD细胞模型和动物模型中,可显著降低Aβ表达,减少Aβ斑块沉积,并抑制星形胶质细胞活化,恢复AD模型中神经元记忆和突触可塑性相关基因的表达。Zavatti等^[77]^还发现,人羊水干细胞外泌体能够显著减少Aβ和小胶质细胞共同作用下的神经元凋亡,减轻小胶质细胞引起的炎症损伤和氧化应激,修复神经元损伤。此外,将辅酶Q10装载到脂肪源性干细胞外泌体后注射到AD大鼠模型体内,能够进一步提高辅酶Q10的治疗效果,从而改善AD相关的炎症和氧化应激反应^[78]^。

在AD模型中,经过热休克处理的神经干细胞外泌体能够有效抵御氧化应激及Aβ诱导的神经毒性^[79]^。此外,M2型小胶质细胞来源的外泌体也表现出降低Aβ斑块形成、减少Aβ寡聚体表达的效果^[80]^。研究显示,装载姜黄素和槲皮素等药物的外泌体与游离药物相比能够更有效地穿过血脑屏障,抑制Tau蛋白的磷酸化和神经纤维缠结的形成,减少神经元死亡,有效改善AD小鼠的认知功能障碍^[81]^。另外,一些装载特定miRNA的外泌体还能够通过抑制Aβ的形成、Tau蛋白的磷酸化及神经炎症来改善AD患者的认知功能,如转染miR-124-3p的小胶质细胞外泌体已被证实能有效减轻神经元的退行性病变^[27]^。

### 4.3 外泌体与帕金森病的治疗

目前PD的治疗与AD相似,主要以对症支持为主,缺乏阻止疾病进展的有效手段,且血脑屏障限制了许多治疗的应用。研究发现,通过外泌体装载的过氧化氢酶在PD的体外模型中可被神经元有效吸收,保护过氧化氢酶免受蛋白酶降解。Hall等^[82]^的研究显示,外泌体携带的过氧化氢酶能有效降低巨噬细胞中的活性氧水平。分离自多巴胺能神经元分化期间的外泌体可显著减轻PD大鼠模型的神经炎症和神经损伤,通过重塑炎症微环境和修复神经损伤来逆转PD病理特征^[83]^。

MSC-Exos能够增强人脑微血管内皮细胞的血管生成,改善PD小鼠模型的运动功能,减少神经元损失,并降低炎症因子水平^[84]^,从而显示了外泌体在神经保护和血管生成治疗领域的前景。Qu等^[85]^研究发现,血液外泌体能够穿过血脑屏障,并通过转铁蛋白与受体间的相互作用将携带的多巴胺高效传递至大脑,使大脑中多巴胺分布增加超过15倍。在PD小鼠模型中,与左旋多巴或游离多巴胺治疗相比,负载多巴胺的外泌体静脉注射后全身毒性更低,且在改善多巴胺能神经元功能和PD小鼠行为症状方面的效果更显著。

同样地,负载姜黄素的人子宫内膜干细胞来源的外泌体能够有效穿透血脑屏障,通过提高抗凋亡蛋白BCL2的表达水平,降低与细胞凋亡过程密切相关的蛋白BAX和Caspase 3的表达,抑制多巴胺能神经元的变性,减少α-syn的聚集,从而改善PD小鼠的运动协调能力^[86]^。Ren等^[87]^还通过修饰外泌体,使其携带神经元特异性的狂犬病病毒糖蛋白(RVG)肽,成功地将DNA适配体传递至神经元,显著减少了由α-syn诱导的病理聚集和突触蛋白丢失。研究人员通过腹腔注射RVG修饰的外泌体,有效减少了PD小鼠纹状体内α-syn的病理聚集,并改善了运动障碍。此外,研究表明,下调外泌体中的miR-137能够通过上调抗氧化基因1 (oxidation resistance gene 1, *OXR1*),有效减轻PD中的氧化应激损伤。在PD小鼠模型中,miR-137下调表现为爬杆时间缩短、牵引测试评分提高、神经元活力增强、凋亡减少以及丙二醛和活性氧水平降低、超氧化物歧化酶水平升高^[88]^。

尽管外泌体载药治疗PD的研究取得了一定进展,但大多仍停留在动物实验阶段,临床试验面临诸多挑战,如缺乏成熟的纯化技术、产量低、成本高、潜在不良反应等,仍需进一步研究。因此,推进临床试验以应用外泌体治疗PD是未来研究的重点和关键突破点。

### 4.4 外泌体与HD、ALS的治疗

外泌体在治疗HD和ALS方面展现出独特的潜力。研究表明外泌体可作为递送治疗性RNA和蛋白质等的优良载体,跨越血脑屏障,为神经退行性疾病的治疗提供了一条新途径。Lee等^[89]^的研究表明miR-124的下调可能导致神经细胞功能和存活相关基因失调,miR-124可以通过外泌体递送,在HD模型中抑制病理性基因*REST*的表达,促进神经元分化和存活,减缓疾病进展。HD患者*HTT*基因第一个外显子过表达时,外泌体miR-34b水平显著升高,而抑制miR-34b可减少HTT蛋白在细胞中的分布并减轻其体外毒性^[90]^。脂肪来源干细胞分泌的外泌体能够通过激活过氧化物酶体增殖物激活受体γ辅激活因子1α通路(PGC-1α)来减少HTT蛋白聚集物的积累^[91]^,这为HD的治疗提供了潜在方法。Di-diot等^[92]^将疏水性修饰的小干扰RNA装载至外泌体中,这些外泌体能够被小鼠原代皮质神经元有效内化,从而实现了对HTT蛋白mRNA和蛋白的剂量依赖性沉默。

Lee等^[91]^研究发现,脂肪来源干细胞释放的外泌体能减少突变SOD 1在G93A神经元细胞中的聚集,而Bonafede等^[93]^进一步证实了脂肪来源干细胞分泌的外泌体能保护NSC-34细胞免受氧化损伤并提高细胞活力,展现出治疗ALS的潜力。总体而言,外泌体在递送治疗性RNA、蛋白质和其他生物活性分子方面展示了很大的治疗潜力。然而,外泌体在神经退行性疾病治疗的临床应用方面仍面临技术挑战,包括外泌体的高效生产、纯化以及治疗靶向性优化等问题。

## 5 总结与展望

在神经退行性疾病的研究领域,外泌体扮演着重要角色。它们通过携带蛋白质、mRNA或miRNA等成分,影响受体细胞基因表达和相关蛋白致活性,并参与病理性蛋白质的传播,通过调控神经炎症和病理蛋白沉积等途径参与疾病的发生和发展。此外,外泌体的成分具有特异性,能够反映其细胞来源和病理状态,因此有望成为神经退行性疾病早期诊断、病情监测和疗效评估的生物标志物。另一方面,外泌体因其能够穿越血脑屏障,其双层脂质膜可以保护内含物免于降解,并且自体来源的外泌体不易引发免疫排斥反应,使其成为中枢神经系统疾病治疗的理想药物递送载体。然而,外泌体在临床应用中依然面临多重挑战,包括如何提高药物载体的有效性与安全性、优化外泌体的提取效率和经济性,以及保证其在体内的稳定性。此外,尚需进一步探讨外泌体的细胞间传递机制、其进入大脑、递送内容物到中枢神经系统的具体机制和效率等。

另外,从体液中分离纯化外泌体是神经系统疾病研究的重要技术步骤。目前,外泌体的分离纯化仍然是制约其应用的技术瓶颈。由于临床样本体积小、成分复杂,外泌体含量较低,特别是脑脊液样本,外泌体分离难度更大。目前常用方法包括差速离心法,简单易行但回收率、纯度有限;密度梯度超速离心法利用密度介质进一步提高纯度,但耗时较长;超滤法依靠滤膜筛选,快速高效但可能造成外泌体破损;沉淀试剂法如ExoQuick^TM^适合高通量处理,但得到的外泌体杂质较多;免疫亲和捕获法可以获得纯度较高的外泌体,但成本高,且仅能得到部分具有相应抗原的外泌体;微流控技术精准高效,适用于小量样本但设备复杂。根据实验需求,可选择单一或组合方法以提高分离效率和纯度。Feng等^[94]^通过在硝酸纤维素膜上涂覆两亲-树状大分子探针(ADSP)实现了外泌体的高效阵列捕获,为外泌体在疾病早期诊断、治疗监测和预后评估等方面的应用提供了高效平台。

为了推进外泌体在神经退行性疾病治疗中的应用,未来研究应深入探讨不同来源外泌体的功能和组成差异,如何实现靶向递送,以及外泌体含量如何影响治疗效果等。这些研究将为外泌体的临床应用提供更坚实的科学依据,推动外泌体在神经退行性疾病治疗中的发展,从而为患者提供更有效的治疗方案。
